# Effects of hydrogen-rich water on abnormalities in a SHR.Cg-*Lepr^cp^*/NDmcr rat - a metabolic syndrome rat model

**DOI:** 10.1186/2045-9912-1-26

**Published:** 2011-11-03

**Authors:** Michio Hashimoto, Masanori Katakura, Toru Nabika, Yoko Tanabe, Shahdat Hossain, Satoru Tsuchikura, Osamu Shido

**Affiliations:** 1 Department of Environmental Physiology, Shimane University Faculty of Medicine, Izumo, Shimane 693-8501, Japan; 2 Department of Functional Pathology, Shimane University Faculty of Medicine, Izumo, Shimane 693-8501, Japan; 3 Department of Biochemistry and Molecular Biology, Jahangirnagar University, Savar, Dhaka 1342, Bangladesh; 4 Disease Model Cooperative Research Association, Hamamatsu, Shizuoka 433-8114, Japan

**Keywords:** hydrogen-rich water, renal glomerulosclerosis, metabolic syndrome model rats, oxidative stress

## Abstract

**Background:**

Hydrogen (H_2_), a potent free radical scavenger, selectively reduces the hydroxyl radical, which is the most cytotoxic of the reactive oxygen species (ROS). An increase in oxygen free radicals induces oxidative stress, which is known to be involved in the development of metabolic syndrome. Therefore, we investigated whether hydrogen-rich water (HRW) affects metabolic abnormalities in the metabolic syndrome rat model, SHR.Cg-*Lepr^cp^*/NDmcr (SHR-cp).

**Methods:**

Male SHR-cp rats (5 weeks old) were divided into 2 groups: an HRW group was given oral HRW for 16 weeks, and a control group was given distilled water. At the end of the experiment, each rat was placed in a metabolic cage for 24 h, fasted for 12 h, and anesthetized; the blood and kidneys were then collected.

**Results:**

Sixteen weeks after HRW administration, the water intake and urine flow measured in the metabolic cages were significantly higher in the HRW group than in the control group. The urinary ratio of albumin to creatinine was significantly lower and creatinine clearance was higher in the HRW group than in the control group. After the 12-h fast, plasma urea nitrogen and creatinine in the HRW group were significantly lower than in the control group. The plasma total antioxidant capacity was significantly higher in the HRW group than in the control group. The glomerulosclerosis score for the HRW group was significantly lower than in the control group, and a significantly positive correlation was observed between this score and plasma urea nitrogen levels.

**Conclusion:**

The present findings suggest that HRW conferred significant benefits against abnormalities in the metabolic syndrome model rats, at least by preventing and ameliorating glomerulosclerosis and creatinine clearance.

## Background

Hydrogen (H_2_), a potent free radical scavenger, selectively reduces the hydroxyl radical, which is the most cytotoxic of the reactive oxygen species (ROS). In addition, water saturated with H_2 _(H_2_-rich water) (HRW) orally administered to rats reduces oxidative stress in the animals, suggesting the molecule's anti-oxidative potency. Molecular H_2 _reportedly acts as a therapeutic antioxidant by reducing cytotoxic oxygen radicals [1]; however, its beneficial effects on pathophysiological functions remain unknown.

Oxidative stress represents an imbalance between the production of ROS and the activity of the antioxidant defense system. An increase in oxygen free radicals induces oxidative stress, which is known to be involved in the development of metabolic syndrome. Metabolic syndrome is characterized by a cluster of metabolic risk factors for atherosclerosis, including obesity, insulin resistance, hyperglycemia, hyperlipidemia, and hypertension [2-4]. Metabolic syndrome also increases susceptibility to chronic renal disease [5]. Drinking HRW is a potentially novel therapeutic and preventive strategy against metabolic syndrome [6]. Thus, the antioxidative potency of HRW may affect the development of metabolic syndrome.

Here, with the use of the SHR.Cg-*Lepr^cp^*/NDmcr (SHR-cp) rat, a metabolic syndrome rat model, we investigated whether HRW affects the rats' metabolic abnormalities. SHR-cp rats spontaneously develop obesity, hypertension, hyperlipidemia, hyperglycemia, and hyperinsulinemia, i.e., metabolic syndrome [7]. The syndrome is comprised of several risk factors for organ damage that operate at high levels of intensity [8]. Thus, this rat model appears well suited for assessing the renal changes induced by broad metabolic abnormalities and the development of glomerular damage such as focal and segmental glomerulosclerosis.

## Materials and methods

### Animals

Male SHR-cp rats (5 weeks old) supplied by the Disease Model Cooperative Research Association (Kyoto, Japan) were randomly divided into 2 groups: an HRW group (n = 12) was given oral HRW for 16 weeks, and a control group (n = 12) was given distilled water. Nakao *et al*. have described the production and characterization of HRW [6]. HRW was prepared by dipping a plastic-shelled product (stick) consisting of metallic magnesium (99.9% pure) and natural stones (Doctor SUISOSUI^®^; Friendear Inc., Tokyo, Japan) into distilled water. HRW was freshly prepared every other day in a 200-mL bottle containing the stick, and the H_2 _concentration was maintained between 0.3 and 0.4 ppm during the experiment. The HRW contained 23 mg/L of calcium, 5 mg/L of magnesium, 19 mg/L of sodium, less than 1 mg/L of potassium and a pH of 7.2. SHR-cp rats were housed in an air-conditioned animal room with a 12:12-h dark:light cycle under controlled temperature (23 ± 2°C) and humidity (50 ± 10% relative humidity). They were given free access to a Quick Fat diet (CLEA Japan Inc., Tokyo, Japan) and a bottle containing either HRW or distilled water. The water intake of the rats was measured every 2 days. All animal experiments were carried out in accordance with the procedures outlined in the Guidelines for Animal Experimentation of Shimane University compiled from the Guidelines for Animal Experimentation of the Japanese Association for Laboratory Animal Science.

### Urine and blood collection

After 16 weeks of HRW ingestion, each rat was weighed and placed in a metabolic cage for 24-h urine collection. Following this, the rat was fasted for 12 h and anesthetized with intraperitoneal sodium pentobarbital (65 mg/kg); its blood was then collected and its kidneys excised.

### Biochemical measurements in blood and urine

Plasma total cholesterol, triglycerides, glucose, creatinine and blood urea nitrogen (BUN) concentrations were determined with an automatic analyzer (BiOLiS 24i; Tokyo Boeki Medical System Ltd., Tokyo, Japan). The concentration of 8-hydroxy-2'-deoxyguanosine (8-OHdG) in plasma was determined by enzyme immunoassay (Highly Sensitive 8-OHdG Check; Japan Institute for the Control of Aging, Shizuoka, Japan). The plasma total antioxidant capacity levels were determined by the biological antioxidant potential (BAP) test (Free Radical Analytical System 4; H&D srl, Parma, Italy). The BAP measurement is based on the ability of a colored solution containing a source of ferric (Fe^3+^) ions adequately bound to a special chromogenic substrate (thiocyanate derivative) to discolor when Fe^3+ ^ions are reduced to ferrous ions (Fe^2+^) in response to the reducing activity of blood samples [9]. Urine albumin and creatinine levels were measured using the Nephrat kit for the quantitation of rat urinary albumin and the Creatinine Companion kit (Exocell, Philadelphia, PA) according to the manufacture's instructions. The ratio of the concentrations of albumin to creatinine (AC ratio) in urine was used as an index of urinary albumin excretion. Endogenous creatinine clearance (CrCl) was determined as CrCl = Ucr × V × Pcr^-1^, where Ucr and Pcr are urinary and plasma creatinine concentrations, respectively, and V is urine flow. The Ucr and V values were calculated from the data of SHR-cp rats in metabolic cages, and Pcr values were cited from Table 1. The CrCl was used as an index of glomerular filtration rate (GFR).

**Table 1:** Biochemical parameters of plasma

	Control group (n = 12)	HRW group (n = 12)
Triglyceride (mg/dL)	443.9 ± 34.5	548.8 ± 50.4
Total cholesterol (mg/dL)	151.3 ± 4.8	153.3 ± 7.6
Glucose (mg/dL)	217.0 ± 35.8	229.2 ± 45.6
BUN (mg/dL)	24.0 ± 0.7	20.9 ± 0.7*
Creatinine (mg/dL)	0.25 ± 0.02	0.20 ± 0.01*
BAP (μmol/L)	2148 ± 91.6	2620 ± 159*
8-OHdG (μg/mL)	0.266 ± 0.02	0.250 ± 0.01

BAP, biological antioxidant potential; BUN, blood urea nitrogen; HRW group; rats orally administered with hydrogen-rich water; 8-OHdG, 8-hydroxy-deoxyguanosine.

At the end of this study, each rat was weighed and placed in a metabolic cage for 24-h urine collection. After urine collection, the rat was fasted for 12 h and anesthetized, and its blood was collected. Values represent mean ± SE. *P < 0.05.

### Morphological analysis

Coronal sections of renal tissue (3-4 μm thick) were stained with periodic acid-Schiff (PAS) and examined by light microscopy in a blinded fashion. Glomerulosclerosis was semi-quantitatively evaluated according to criteria developed by Uehara *et al *[10]. Briefly, 50 glomeruli were randomly selected from each animal for morphometric analysis. Glomerulosclerosis, defined as synechiae formation by PAS staining with focal or global obliteration of capillary loops, was graded as follows: 1+, < 30% of glomerular area affected; 2+, 30% to 70% affected and 3+, > 70% affected. The overall glomerulosclerosis score per animal was the average grade of all the glomeruli evaluated.

### Statistical analysis

All data are expressed as the means ± SE. Significant differences between HRW and control groups were determined by the unpaired Student's *t*-test. Correlation was determined by Pearson's correlation analysis. Differences of P < 0.05 were considered significant. PASW Statistics 18 was used for the statistical analysis (SPSS Inc., Chicago, IL, USA).

## Results

### Body weight and HRW intake

**Figure 1:**
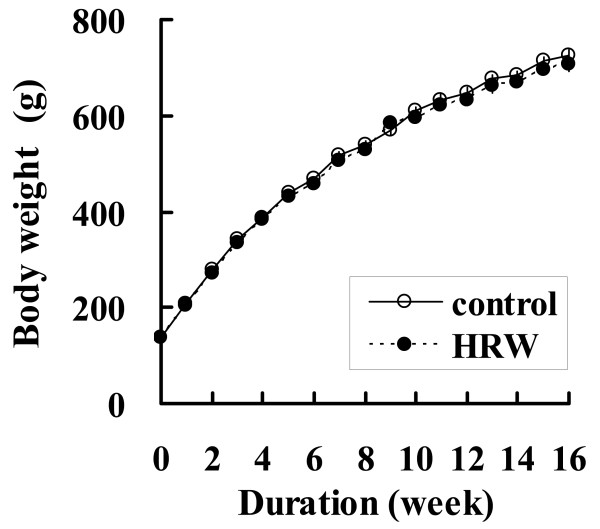
**Effect of HRW administration on body weight**. White circle, distilled water-administered rats (control, n = 12); black circle, hydrogen-rich water-administered rats (HRW, n = 12). *P < 0.05.

HRW administration did not affect the body weight of SHR-cp rats throughout the experimental period (Figure 1). The volume of water intake per 24 h measured in the metabolic cages was larger in the HRW group than in the control group (Table 2).

**Table 2:** Effects of hydrogen-rich water (HRW) on water intake and renal functions in SHR-cp rats

	Water intake	Urine flow	Urine			Creatinine clearance
				
			Albumin (A)	Creatinine (C)	AC ratio	
	(mL/kg BW. day)	(mL/kg BW. day)	(mg/kg BW. day)	(mg/kg BW. day)		(mL/min)
Control group	71.1 ± 1.8	47.5 ± 2.4	161.7 ± 14.0	24.4 ± 1.3	6.81 ± 0.64	5.16 ± 0.4
(n = 10)						
HRW group	113.7 ± 3.5*	62.0 ± 6.3*	129.9 ± 10.9	25.1 ± 0.6	5.17 ± 0.42*	6.28 ± 0.36*
(n = 11)						

Values are mean ± SE. *P < 0.05, BW, body weight; AC ratio, albumin to creatinine ratio.

These data were obtained from SHR-cp rats housed for 24 h in metabolic cages, except that of plasma creatinine concentrations, which were cited in Table 1.

### Plasma biochemical data, water intake, and parameters of renal functions

The plasma biochemical data in the control and HRW rats fasted for 12 h after 16 weeks are listed in Table 1. Plasma BUN and creatinine concentrations were significantly lower in the HRW group than in the control group. There was no significant difference in the concentrations of plasma triglyceride, total cholesterol, or the level of plasma 8-OHdG between the 2 groups; however, the plasma BAP level in the HRW group was significantly higher.

The water intake and renal function parameters measured in the metabolic cages at 16 weeks of HRW administration are listed in Table 2. Water intake and urine flow measured for 24 h in the metabolic cages were significantly higher in the HRW group than in the control group. Urine albumin was lower, but not significantly so, in the HRW group than in the controls (0.05 < P < 0.1), leading to an albumin to creatinine ratio of 24.1% in the HRW group that was significantly lower than that in the control group. CrCl increased with HRW administration in SHR-cp rats, with a 21.7% potentiation compared with the control group.

### Effect of HRW administration on glomerular sclerosis

**Figure 2:**
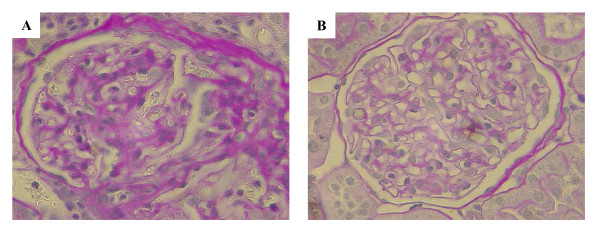
**Photomicrographs of coronal sections of the glomeruli from SHR-cp rats**. (A) Control group. (B) HRW group. Periodic acid-Schiff (PAS) staining of the control group revealed glomerular damage, which was characterized by segmental glomerular sclerosis and the formation of synechiae by the attachment of parietal epithelial cells to the denuded glomerular basement membrane (PAS stain, original magnification ×400).

**Figure 3:**
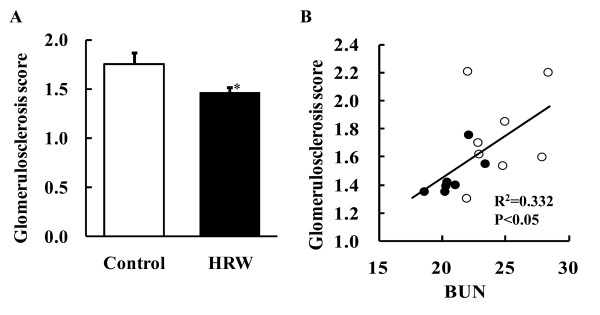
**Effect of HRW administration on glomerular sclerosis**. After 16 weeks of HRW administration, coronal sections of the renal tissue from distilled water-administered rats (control, n = 8) and HRW-administered rats (HRW, n = 7) were stained with PAS and examined by light microscopy in a blinded fashion (Figure 3A). Glomerulosclerosis was semi-quantitatively evaluated according to the criteria developed by Uehara et al. [10]. *P < 0.05. Data represent mean ± SE. Figure 3B illustrates the relationship between the glomerulosclerosis score and concentrations of blood urea nitrogen. White and black circles indicate control and HRW rats, respectively.

HRW administration inhibited histological damage to the kidneys of SHR-cp rats (Figure 2). The glomerular sclerosis score was significantly lower in the HRW group (1.46 ± 0.06) than in the control group (1.75 ± 0.11) (Figure 3A). Simple regression analyses were performed to determine whether an alteration in the glomerulosclerosis score was associated with plasma BUN and other parameters used as indices of kidney damage. A significantly positive correlation was observed only between the glomerulosclerosis score and plasma BUN levels (Figure 3B), while correlation of the former with other variables such as water intake, urine flow, and CrCl was not statistically significant.

## Discussion

The hydroxyl radical and peroxynitrite are the strongest oxidants that react indiscriminately with nucleic acids, lipids, and proteins, resulting in DNA fragmentation, lipid peroxidation, and protein inactivation. Molecular H_2 _reduces the hydroxyl radical and peroxynitrite *in vitro *and induces therapeutic antioxidant activity in the rat middle cerebral artery occlusion model [1]. HRW ingestion reduces oxidative stress in human subjects with potential metabolic syndrome, suggesting that HRW represents a potentially novel therapeutic and preventive strategy for metabolic syndrome [6]. Oxidative stress represents an imbalance between the production of ROS and the activity of the antioxidant defense system. Cardinal *et al*. reported that both local and systemic concentrations of H_2 _measured in the kidneys and serum following oral administration of HRW peaked within 15 min after ingestion, proving that HRW is an effective mode of delivery for H_2 _[11]. The continuous incorporation of H_2 _from the stomach into the blood may alter the state of blood components to a reductive one. Indeed, the plasma BAP levels of SHR-cp rats in the HRW group were significantly higher than that in the control group (Table 1) in this study. Therefore, continuous exposure to H_2 _may influence the oxidative state in organ tissues.

Light microscopy has shown that SHR-cp rats develop glomerular damage, mesangial expansion, and focal and segmental glomerular sclerosis; thus, the glomerulosclerosis score in SHR-cp rats is higher than that in Wistar Kyoto (WKY) rats [8]. In the present study, the glomerulosclerosis score in the HRW group was lower than that in the control group (Figure 3A), suggesting a preventive effect of HRW administration on the development of histologically evident glomerular injury observed in the SHR-cp rats. Increases in plasma creatinine and/or BUN levels were considered indices of damage to renal function. Indeed, the BUN level in SHR-cp rats is 1.65 times greater than that in WKY rats [8]. In this study, HRW administration decreased the plasma BUN and creatinine levels of the SHR-cp rats compared with those of the control rats (Table 1). The HRW administration-induced decreases in plasma BUN and creatinine levels were consistent with the results recently reported by Nakashima-Kaminura *et al *[12]. They reported that HRW prevented metamorphosis-associated decreased apoptosis in the kidney and nephrotoxicity as assessed by serum creatinine and BUN levels. Moreover, HRW ingestion significantly decreases plasma creatinine levels in human subjects with potential metabolic syndrome [6]. These results suggest that continuous HRW administration appears to prevent and ameliorate histological damage to the kidneys.

Recent studies have indicated that metabolic syndrome increases susceptibility to chronic kidney disease [5]. Glomerular and tubulointerstitial damage characteristic of human type II diabetic nephropathy (e.g., focal and segmental glomerular sclerosis) develops in SHR-cp rats together with evidence of increased oxidative stress [13]. In this study, continuous administration of HRW did not affect the body weight or plasma levels of triglycerides, total cholesterol, or glucose in SHR-cp rats, but significantly inhibited the deterioration of glomerulosclerosis. Continuous HRW administration also decreased the urinary AC ratio, which can be used to diagnose the early stages of diabetic nephropathy in patients with diabetes [14]. In clinical practice, the measurement of CrCl remains the most widely used method for obtaining a GFR index. SHR-cp rats develop progressive diabetic nephropathy with severe proteinurial and histological abnormalities, which are associated with a decrease in CrCl as compared with WKY rats [15]. In this experiment, HRW administration significantly increased the levels of CrCl in SHR-cp rats (Table 2). These results suggest that the intake of HRW inhibited renal dysfunction in metabolic syndrome model rats. The mechanisms of the increased water intake and urine flow in HRW-administered SHR-cp rats remain to be elucidated. From the present data, it is difficult to clarify the possible causes and consequences of these increments. Typically, urine flow, urinary flow of creatinine, and/or CrCl as GFR indices are multifactorial phenomena. The increase in GFR and the decrease in the AC ratio observed in HRW-administered SHR-cp rats suggest that continuous HRW administration inhibits the development of renal dysfunction, leading to the increased urine flow and, presumably, the increased water intake. Further experiments are required to confirm this.

From the data obtained in this study, it is difficult to clarify the mechanisms underlying the beneficial effects of HRW on renal diseases. The HRW administration-induced increase in water intake, urine flow, and CrCl, and/or the decrease in oxidative stress observed in this study may play a role in this ameliorating effect in SHR-cp rats. Cardinal *et al*. recently reported that oral HRW administration prevents chronic allograft nephropathy after renal transplantation via the ability of molecular H_2 _to reduce oxidative stress-induced damage [11]. Antioxidant enzymes do not detoxify the hydroxyl radical and peroxynitrite, which are target oxidants of molecular H_2_, because no enzyme detoxifies these radicals. H_2 _therapy reduces apoptosis by suppressing caspase activity in the neonatal hypoxia-ischemia rat model [16]. It is also reported that a sufficient supply of H_2_-rich pure water may prevent or delay the development and progression of type II diabetes mellitus by providing protection against oxidative stress [17]. Therefore, our studies suggest that HRW may have direct effects on kidney function and that its administration appeared to ameliorate glomerular damage in a rat model of metabolic syndrome, possibly by limiting oxidative stress. Further studies are needed to confirm these mechanisms.

## Conclusions

The present study was designed to evaluate whether HRW ingestion would have an ameliorative effect on a host of metabolic abnormalities, including glomerulosclerotic damage, blood creatinine and BUN levels, oxidative potentials, urinary flow, and GFR in metabolic syndrome model rats. Based on the biochemical and renal parameter results and morphological changes in the kidneys, the present study clearly indicates that HRW conferred significant benefits against these abnormalities in metabolic syndrome model rats.

## List of abbreviations

The abbreviations used are: AC ratio: ratio of the concentrations of albumin to creatinine; BAP: biological antioxidant potential; BUN: blood urea nitrogen; CrCl: creatinine clearance; GFR: glomerular filtration rate; HRW: hydrogen-rich water; 8-OHdG: 8-hydroxy-2'-deoxyguanosine; PAS: periodic acid-Schiff; ROS: reactive oxygen species; SHR-cp: SHR.Cg-*Lepr^cp^*/NDmcr; WKY: Wistar Kyoto.

## Competing interests

The authors declare that they have no competing interests.

## Authors' contributions

MH, MK, and YT carried out experiments. MH, TN, ST, and OS participated in the design of the study. MK and YT performed the statistical analysis. MH and SH wrote the manuscript. All authors read and approved the final manuscript.
